# Inosine modifications in human tRNAs are incorporated at the precursor tRNA level

**DOI:** 10.1093/nar/gkv277

**Published:** 2015-04-27

**Authors:** Adrian Gabriel Torres, David Piñeyro, Marta Rodríguez-Escribà, Noelia Camacho, Oscar Reina, Adélaïde Saint-Léger, Liudmila Filonava, Eduard Batlle, Lluís Ribas de Pouplana

**Affiliations:** 1 Institute for Research in Biomedicine (IRB Barcelona), C/Baldiri Reixac 10, Barcelona, 08028 Catalonia, Spain; 2 Catalan Institution for Research and Advanced Studies (ICREA), P/Lluis Companys 23, Barcelona, 08010 Catalonia, Spain

## Abstract

Transfer RNAs (tRNAs) are key adaptor molecules of the genetic code that are heavily modified post-transcriptionally. Inosine at the first residue of the anticodon (position 34; I34) is an essential widespread tRNA modification that has been poorly studied thus far. The modification in eukaryotes results from a deamination reaction of adenine that is catalyzed by the heterodimeric enzyme adenosine deaminase acting on tRNA (hetADAT), composed of two subunits: ADAT2 and ADAT3. Using high-throughput small RNA sequencing (RNAseq), we show that this modification is incorporated to human tRNAs at the precursor tRNA level and during maturation. We also functionally validated the human genes encoding for hetADAT and show that the subunits of this enzyme co-localize in nucleus in an ADAT2-dependent manner. Finally, by knocking down HsADAT2, we demonstrate that variations in the cellular levels of hetADAT will result in changes in the levels of I34 modification in all its potential substrates. Altogether, we present RNAseq as a powerful tool to study post-transcriptional tRNA modifications at the precursor tRNA level and give the first insights on the biology of I34 tRNA modification in metazoans.

## INTRODUCTION

Transfer RNAs (tRNAs) are essential molecules required for decoding messenger RNAs (mRNAs) into proteins. They contain a nucleotide triplet (anticodon) composed of residues 34, 35 and 36 of the tRNA molecule that specifically recognizes nucleotide triplets (codons) on mRNAs. tRNAs need to be heavily modified post-transcriptionally in order to become fully active. Modifications in the main body of the tRNA are important for the structure and stability of the tRNA. Modifications at the acceptor stem serve as identity elements for aminoacyl tRNA synthetases, the enzymes that charge the tRNA with their respective amino acid. Modifications at the anticodon arm are usually seen as enhancers of the efficiency and fidelity of mRNA decoding (1). Within the anticodon region, the residue at position 34 of the tRNA (the first nucleotide of the anticodon) is frequently modified to allow the base 34 to ‘wobble’ and pair with non-canonical bases, therefore allowing certain tRNAs to recognize more than one mRNA codon (2).

Inosine is a post-trancriptional modification found at three different positions in tRNAs: position 34, 37 and 57 (for a recent review see (3)). It is the result of a deamination reaction of adenines that is catalyzed by adenosine deaminases acting on tRNAs (ADATs). Inosine 57 is only present in Archaea as 1-methylinosine (m^1^I57) and both its function and the enzyme involved are currently unknown (4). Inosine at position 37 is present only in eukaryotic tRNA^Ala^, where it is also further modified into 1-methylinosine (m^1^I37). The modification is catalyzed by the homodimeric enzyme ADAT1. In yeast, knockouts (KO) of ADAT1 are viable suggesting that m^1^I37 is not an essential tRNA modification (5). However, ADAT1 KO plants showed less biomass when grown under environmental stress (6).

Inosine at position 34 (I34) is present in bacterial tRNA^Arg^ and in 7–8 different eukaryotic tRNAs. The modification is catalyzed by the homodimeric enzyme TadA in bacteria and, in eukaryotes, by the heterodimeric enzyme ADAT (hetADAT), which is composed of two subunits: ADAT2 and ADAT3 (Tad2 and Tad3 in yeast, respectively) (3). ADAT2 is considered the catalytic subunit while ADAT3 might be playing a role in tRNA substrate recognition (7).

While A34 can in principle only pair with codons having a U at the third codon position (U-ended codons); I34 can pair with U-, C- and A-ended codons (2). Moreover, analyses of the tRNA gene copy number in different species revealed that tRNAs with G34 are always absent in genomes that code for tRNAs with A34. This suggests that I34 tRNAs are required to decode C-ended codons (i.e. the cognate codons of G34 tRNAs) in those species (8). The genomic enrichment in tRNAs with A34 in eukaryotes directly correlates with the emergence of heterodimeric ADATs in the same species, indicating that the activity of hetADAT was an important influence in the evolution of eukaryotic genomes (9).

While I34 has been known for several years, very little is known about its exact functional role and the biogenesis of this modification (3). Most of the published work related to this modification has focused on the biochemistry of the deamination reaction and on the tRNA sequence determinants for hetADAT activity *in vitro* (10–15). *In vivo*, eukaryotic I34 and hetADAT have only been characterized in yeast (7,16), *Trypanosoma brucei* (17–19) and recently in *Arabidopsis thaliana* (20). In humans, the I34 modification has been associated to myositis, intellectual disability and strabismus (reviewed in (3)). However, despite the increasing links between tRNA modifications and human physiology, and the growing number of associations between dysregulation of tRNA modifications and human diseases (21), no characterization of metazoan hetADAT has been reported to date.

In this work, we decided to use a human cell line to give the first insights into hetADAT and the I34 modification in metazoans. Using high-throughput small RNA sequencing (RNAseq), we investigate at which step during the biogenesis of tRNAs the I34 modification occurs. We also functionally validate the annotated human genes encoding for ADAT2 and ADAT3 proteins and investigate their subcellular localization. Finally, we knocked down HsADAT2 and show for the first time that it is possible to modulate the levels of I34 modification on all eight human tRNA substrates of hetADAT and that these fluctuations are tolerated by the cell.

## MATERIALS AND METHODS

### Cell lines and cell culture

Human Embryonic Kidney 293T (HEK293T) cells were used to generate stably expressing short-hairpin RNA (shRNA) cell lines by lentiviral infection as described previously (22). Vectors containing the shRNA sequences were obtained from the Human MISSION shRNA library (Sigma): MISSION pLKO.1-puro non-mammalian shRNA control plasmid DNA (shCV; cat number: SHC002) and ADAT2 MISSION shRNA plasmid DNA (shADAT2; cat number: SHCLND-NM_182503; clone id: TRCN0000050656). Cells were maintained at 37°C/5% CO_2_ in Dulbecco's Modified Eagle's Medium containing 10% fetal bovine serum, 100 U/mL Pen/Strep and 2 μg/ml puromycin.

### High-throughput small RNA sequencing

All RNAseq experiments were performed in independent duplicates. Total RNA from HEK293T shCV and HEK293T shADAT2 cells was extracted with TRIzol Reagent (Ambion) following the manufacturer's protocol. The obtained RNA was ethanol-reprecipitated and resuspended in DEPC-treated water. RNA was quantified using a Nanodrop ND-1000 and the quality of the RNA sample was assessed using a 2100 Bioanalyzer. RNA libraries were performed with the NEBNext Multiplex Small RNA Library Prep Set for Illumina (NEB #E7300S) following the manufacturer's protocol. Samples were loaded in a 3% agarose gel and fragments between 160 and 220 bp (expected size of tRNA molecules plus sequencing adaptors) were selected. Sequencing was performed in a HiSeq2000 SR100 Illumina sequencer (at the CSF NGS Unit, Vienna) running four samples per lane. Around 160 million reads were obtained per lane resulting in around 40 million reads per sample. The RNAseq data generated for this study has been deposited at the EBI European Nucleotide Archive (Accession number PRJEB8019).

### RNAseq analyses and statistical analyses

Solexa Illumina RNAseq data was aligned against the human reference genome hg19 with Bowtie2 version 2.2.2 using local alignment, allowing for one mismatch per seed and using default options. Not aligned terminal bases were soft clipped using the bamutils utility from the NGSutils software version 0.5.7. In order to account for the repetitive nature of tRNA types across the genome, all reads, including those that could map to more than one position, were considered in the analysis. However, read IDs aligning to two or more different tRNA types were identified by using a second round of alignment with Bowtie2, now reporting multiple hits and were removed from the results of the first alignment in order to keep only those reads aligning unequivocally to multiple copies of the same tRNA type. Finally, tRNA copies with a coverage depth below 10 reads at the anticodon position were also removed from the analysis.

Read counts and proportions by feature type (Table 1) were computed in R version 3.0.2 using the *Homo sapiens* hg19 annotation downloaded from Ensembl (October 2014) (23,24). Human hg19 predicted tRNAs were downloaded from the Genomic tRNA database (http://gtrnadb.ucsc.edu) (May 2014) (25). Reads were imported in R using the Rsamtools package version 1.14.3 and classified as either ‘Processed’ tRNAs if they were aligned within the predicted tRNA sequence coordinates or ‘Precursor’ tRNAs if they also overlapped part of the 5′-leader or 3′-trailer genomic sequence.

**Table 1.** RNAseq mapping analysis for all the obtained sequencing reads indicating the proportion (%) of reads mapping to a particular gene biotype

Gene biotype	Average (%) reads	STDEV (%) reads
Processed transcript	37.24	1.02
snoRNA	26.86	0.78
Protein coding	26.55	0.70
Antisense	4.85	0.31
lincRNA	1.19	0.11
tRNA	0.98	0.28
snRNA	0.65	0.13
rRNA	0.61	0.18
mt-tRNA	0.25	0.02
Misc RNA	0.25	0.05
Pseudogene	0.18	0.03
mt-rRNA	0.14	0.02
Sense overlapping	0.14	0.01
Sense intronic	0.08	0.01
miRNA	0.05	0.01

Shown are the average and standard deviation from four independent RNAseq experiments. Gene biotypes used are those defined by GENCODE (24).

**Figure 1.**
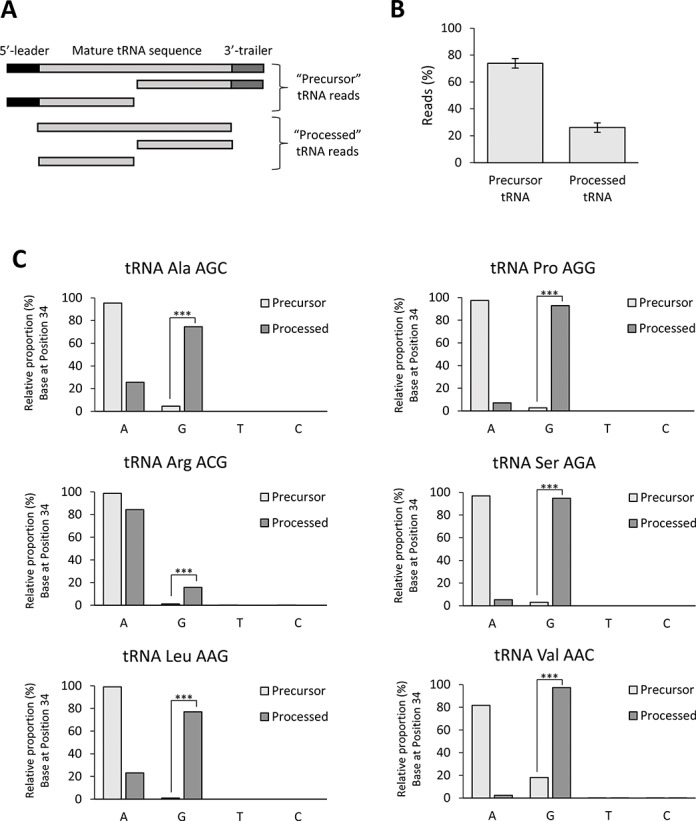
(**A**) Schematic representation of the structure of tRNA reads detected in this study. The two defined groups of sequences (Precursor tRNA and Processed tRNA) are shown. (**B**) Proportion (%) of reads distributed in the Precursor tRNA and Processed tRNA groups. (**C**) Analysis of observed bases at position 34 for ANN tRNAs in the Precursor tRNA and Processed tRNA groups. Statistical significance was obtained by Fisher Exact Test with Benjamini–Hochberg adjusted *P*-values (***: *P*-value ≤ 0.001).

Absolute genomic position coordinates for the anticodon of predicted human hg19 tRNAs were determined with R and base calling at 50 bp up and downstream of the anticodon was performed using IGVTools version 2.2.23. Base calling results for individual tRNA type copies in shCV and shADAT2 replicates were aggregated using custom R scripts. Benjamini–Hochberg adjusted *P*-values for statistical significance of A/G mismatch proportion between ‘Precursor’ and ‘Processed’ tRNAs (Figure 1B) and between shCV and shADAT2 cells (Figure 4B) were computed with the Fisher Exact Test.

### *In vitro* deamination assays

*In vitro* transcribed pre-tRNA^Val^_(AAC)_ was performed as described previously (26) except that the construct containing the tRNA sequence was digested with NspI instead of BstNI before transcribing the tRNA. Oligonucleotides used to assemble the tRNA for cloning into pUC19 vector are described in Supplementary Table S1.

Human ADAT2 and ADAT3 were obtained from the IRB Protein Expression Facility (Nick Berrow, IRB Barcelona) using the protein sequences annotated in NCBI: NP_872309.2 (HsADAT2) and AAH11824 (HsADAT3) as cloning inserts. Human ADAT2 and ADAT3 were cloned into pOPINFS expression vectors (Oxford Protein Production Facility, UK; (27)) as a bicistronic product resulting in HsADAT2 N-term His-tag and HsADAT3 C-term StrepII-tag fusion proteins separated by a ribosome binding site. The construct was expressed in *Escherichia coli* Rosetta strain grown in Overnight Express Instant TB medium (Novagene). The heterodimer (HsADAT2 and HsADAT3) was purified by affinity chromatography with a StrepTrap HP column (GE Healthcare). HsADAT2 and HsADAT3 were purified at an equimolar ratio. Purified hetADAT was stored in 50 mM Hepes pH 8, 100 mM KCl, 1mM MgCl_2_, 0.1 mM ethylenediaminetetraacetic acid, 2 mM dithiothreitol (DTT) with 20% glycerol.

For the deamination assays, 5 μg of *in vitro* transcribed pre-tRNA^Val^_(AAC)_ was incubated with 20 μl of purified hetADAT [1.3 μM] in a final volume of 110 μl (DEPC-treated water) for 1 h at 37°C. Afterwards, an additional 10 μl of hetADAT [1.3 μM] was added and the reactions were further incubated for 1 h at 37°C. Reactions were purified with Viogene miTotal RNA mini kit (Cat number: VTR1002) and RNA was eluted from the purification column with DEPC-treated water and was quantified using a Nanodrop ND-1000. One microgram pre-tRNA^Val^_(AAC)_ (incubated or not with hetADAT) was retro-transcribed (RT) with High Capacity cDNA Reverse Transcription Kit (Part number: 4368814) following the manufacturer's protocol using pre-tRNA^Val^_(AAC)_ oligo 6 (Supplementary Table S1) as RT-primer. RT-cycle: 10 min at 25°C; 60 min at 37°C; 5 min at 85°C. Following the RT-reaction, a standard polymerase chain reaction (PCR) reaction was performed using oligos pre-tRNA^Val^_(AAC)_ 1 and 6 (Supplementary Table S1) as primers forward and reverse, respectively. Two identical 20 μl reactions using 2 μl cDNA for each reaction was performed. PCR cycle: 3 min at 94°C (one cycle); 45 s at 94°C, 30 s at 55°C, 90 s at 72°C (35 cycles); 10 min at 72°C (one cycle). Following the PCR reaction, the two identical PCR reactions were combined and purified using Nucleospin Gel and PCR clean-up kit (Macherey-Nagel; REF number: 740609.250). Samples were sent for sequencing using the same oligos used for the PCR reaction as sequencing primers.

The Endonuclease V (EndoV) assay was performed by incubating 1 μg *in vitro* transcribed pre-tRNA^Val^_(AAC)_ (incubated or not with hetADAT) with 1 μl EndoV (New England Biolabs) in a 50-μl reaction containing buffer 4 (New England Biolabs) and DEPC-treated water, overnight at 37°C. RNA was then dephosphorylated by addition of 1 μl Calf Intestinal Phosphatase (New England Biolabs) to each EndoV reaction and incubation for 1 h at 37°C. Reactions were purified using Viogene purification kit (see above) and were 5′-radiolabelled with γ-^32^P[ATP] following standard procedures in 50 μl reactions. Two microlitre of 5′-radiolabelled RNA was pre-heated at 95°C for 2 min and was ran in a 8% polyacrylamide/8 M urea denaturing gel for 1 h at 120 V. The radioactive gel was directly exposed to a phosphorimager screen and was developed in a Typhoon 8600 Variable Mode Imager (Molecular Dynamics). Radioactive RNA decade marker (Ambion) was used to estimate the size of the tRNA products.

### Confocal microscopy

Human ADAT2 (NCBI: NM_182503.2) and ADAT3 (NCBI: NM_138422.2) for the synthesis of fluorescent-tagged proteins were amplified from cDNA obtained from HEK293T cells using the primers listed in Supplementary Table S1. For the generation of mCherry-tagged proteins, the HTP In-Fusion Cloning system (BD Clontech) was used following the manufacturer's manual. PCR fragments were cloned into pPEU17 (N-term mCherry) or pPEU19 (C-term mCherry) vectors (28). GFP-tagged proteins were generated by cloning of PCR fragments into pEGFP-N1 (C-term eGFP) or pEGFP-C1 (N-term eGFP) vectors (BD Clontech). All constructs were validated by Sanger sequencing.

HEK293T cells were lipofected using Lipofectamine 2000 (Invitrogen) following the manufacturer's protocol. Every lipofection involved a 2-plasmid co-transfection as shown in Figure 3 and Supplementary Figure S7. 1.25 μg total DNA (0.625 μg per plasmid) was lipofected into cells growing in 6-well plate format in a 2.5 ml final volume reactions. Forty-eight hours after transfection, cells were stained with Hoechst 33342 (Sigma; Cat Number: B2261–25MG) and visualized (live imaging) in a Leica TCS SP2 AOBS confocal microscope using a HCX PL APO 63x/1.4–0.6 Oil Lbd BL objective and a 2× digital zoom. Images were scanned in sequential mode for the Hoechst, eGFP and mCherry channels.

### Reverse transcription-quantitative PCR (RT-qPCR)

Five hundred nanograms of total RNA from HEK293T shCV and HEK293T shADAT2 cells were retro-transcribed as mentioned above (see *in vitro deamination assays*) using an OligodT as RT-primer. RT-cycle: 10 min at 25°C; 120 min at 37°C; 5 min at 85°C. Real-Time PCR was performed in a StepOne Plus Real Time PCR System (Applied Biosystems) using SYBR Green PCR Master Mix (Applied Biosystems) following the manufacturer's protocol. Primers used for HsADAT2 amplification are described in Supplementary Table S1. qPCR cycle: 10 min at 95°C (1× cycle); 15 s at 95°C, 30 s at 57°C, 30 s at 60°C (40 cycles; PCR step); 15 s at 95°C, 60 s at 60°C, 15 s at 95°C (one cycle; Melting Curve step).

## RESULTS

### Standard small RNA sequencing as a tool to study pre-tRNAs

It is generally believed that nearly all molecules of natural mature tRNAs bearing an A at position 34 (ANN tRNAs) are deaminated into I34 (29). However, whether this modification occurs at the precursor or the mature tRNA level remains controversial (3).

To address this question we decided to perform standard high-throughput small RNA sequencing (RNAseq) on the commonly used human embryonic kidney 293T (HEK293T) cell line. Inosine is structurally a guanosine (G) analogue and as a result, inosines on nucleic acids are read as a ‘G’ upon sequencing (7,16–20,30,31). Therefore, detecting a G34 on a sequencing read that unequivocally mapped to an ANN tRNA, strongly supports that the A34 has been deaminated into I34.

It is known that in an RNAseq experiment, there is a strong bias against sequencing fully matured tRNAs. This is because they have strong secondary structures, can carry an amino acid at their 3′-end that can affect the ligation of the adaptors necessary for sequencing and are frequently post-transcriptionally modified with chemical modifications that are incompatible with the sequencing reactions (32–34). However, we envisioned that an RNAseq strategy would be suitable to detect precursor tRNAs (pre-tRNA) at different stages of the maturation process. As shown in Table 1, we found about 1% of all the sequencing reads mapping to tRNA sequences, consistent with the known difficulty to sequence this RNA class. We next eliminated the tRNA reads that mapped to more than one type of tRNA and kept those reads that unequivocally mapped to a single tRNA type (see ‘Material and Methods’ section). Within these reads, most of them contained the 5′-leader and/or 3′-trailer sequences present in pre-tRNAs (herein ‘Precursor’ tRNA reads). We also detected tRNA reads lacking leader and trailer sequences (herein ‘Processed’ tRNA reads) that included tRNA fragments that may derive from pre-tRNAs or mature tRNAs and, to a much less extent, full length mature-like tRNA sequences. We were therefore able to have sufficient reads to study pre-tRNAs and to group those sequencing reads into two groups that would reflect two stages of the tRNA maturation: ‘Precursor’ (early precursor) and ‘Processed’ (tRNA sequences after 5′-leader and 3′-trailer removal) (Figure 1A). We found that overall, about 70% of the tRNA reads contained leader and/or trailer sequences while about 30% of the reads were lacking these sequences (Figure 1B); however, these proportions were tRNA type dependent (Supplementary Figure S1).

Over the course of writing this manuscript Pang *et al*. suggested a modification to the small RNAseq protocol that would allow for improved sequencing of fully matured tRNAs (32). Briefly, the authors first ligated 3′-adaptors to the tRNAs, then performed the retrotranscription reaction (RT-reaction) and finally ligated the other adaptors (at 5′-end of the tRNA sequence) to the cDNA products. This is in contrast to the standard RNAseq reactions, like the one used in this study, where 3′- and 5′-adaptors are ligated before the RT-reaction. When using the modified protocol, if a mature tRNA contains a post-transcriptional modification that is incompatible with the RT-reaction, such tRNA will still be amplified by PCR and sequenced as a shorter tRNA read. On the contrary, using the standard RNAseq protocol, such modified mature tRNA would not be sequenced at all because it will lack the 5′-end adaptor necessary for amplifying and sequencing of the product. In light of this information, we are certain that the short tRNA reads that we observed correspond to tRNA fragments and not to a tRNA containing a post-transcriptional modification incompatible with the RT-reaction. Further, it is also very likely that the few sequencing reads we observed for mature-like tRNA sequences lacking 5′-leader and 3′-trailer sequences does not correspond to fully matured tRNAs. We therefore considered our ‘Processed’ tRNA reads as precursor tRNAs further downstream on the tRNA maturation process.

### Inosine modifications occur at the pre-tRNA level

We next evaluated the proportions of A, G, C and T observed at position 34 for every human ANN tRNA. As shown in Figure 1C and Supplementary Figure S2, inosine (detected as ‘G’) could be observed at a low frequency already in the reads corresponding to early precursors in all eight ANN tRNAs. Furthermore, the presence of G34 was even more frequent in the processed tRNA group for six out of the eight tRNA substrates of hetADAT (Figure 1C). We could not observe the same pattern for tRNA^Ile^_(AAT)_ and tRNA^Thr^_(AGT)_ because we did not have enough reads in the processed tRNA group for these tRNA species; however, we could detect G34 on both of these tRNA types in the precursor tRNA group (Supplementary Figure S2). Importantly, we did not detect significant levels of C and T at this position for any of the ANN tRNAs consistent with the exclusive detection of either modified or not modified A34 (Figure 1C and Supplementary Figure S2). Additionally, we confirmed that other adenosines within the anticodon (position 35; A35) that are known to be unmodified were in fact read as A35, as expected (Supplementary Figure S3). Further, we ruled out possible effects of sequencing errors by comparing the A-to-G mismatch proportion at position 34 versus the A-to-G mismatch proportion throughout the rest of the tRNA sequence for each tRNA type (Supplementary Figure S4). Notably, we also observed strong differences in the proportion of G34 detection between different tRNA types (Supplementary Figure S4), probably due to sequencing biases. Therefore, it would not be appropriate to compare with this data the levels of G34 (I34) between different tRNA species (e.g. tRNA^Arg^_(ACG)_ would seem to be less modified than tRNA^Val^_(AAC)_). Altogether, the results show that inosine at position 34 occurs at the pre-tRNA level and there is a clear trend of increased detection of such modification as the precursor tRNAs are being processed into their mature forms.

Similar results were obtained for the inosine modification at position 37 only present in tRNA^Ala^. It is known that A37 is first deaminated into I37 by the enzyme ADAT1 and then I37 is methylated by the tRNA methyltransferase 5 (Trm5) resulting in 1-methylinosine (m^1^I) (35,36). Moreover, while inosines are sequenced as a ‘G’, m^1^I are generally sequenced as a ‘T’ (5,6,37). We analysed tRNA^Ala^_(AGC)_ and found significant levels of G37, but not of T37, in the precursor tRNA group. Further, in the processed tRNA group, higher levels of G37 and T37 could be observed (Supplementary Figure S5). These findings are consistent with the known stepwise process for the m^1^I37 modification and suggest that this modification also occurs at the pre-tRNA level and as the pre-tRNA maturates.

### A precursor tRNA can be a substrate of hetADAT

**Figure 2.**
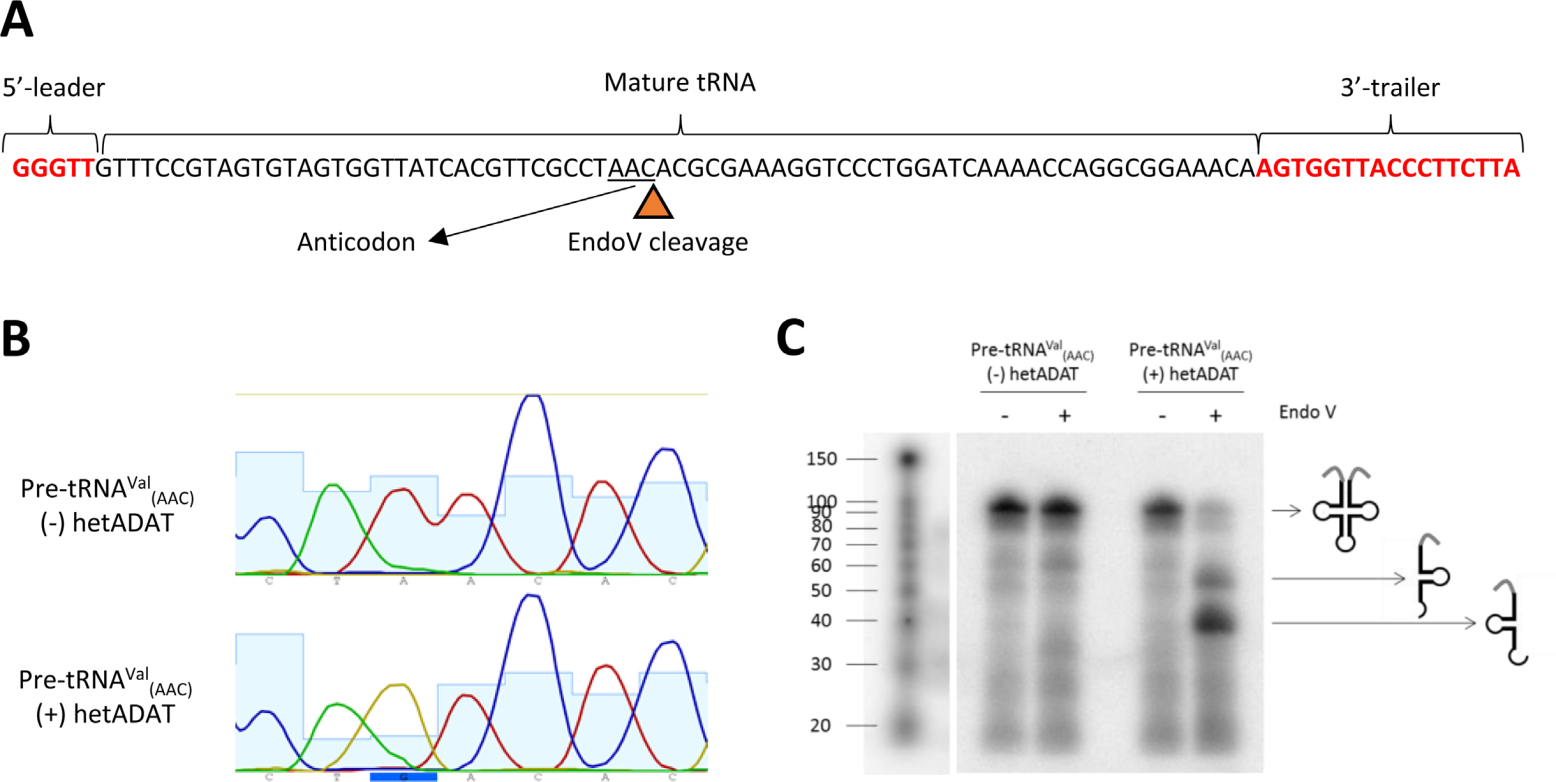
*In vitro* deamination assays. (**A**) Sequence of the *in vitro* transcribed pre-tRNA^Val^_(AAC)_ showing the 5′-leader, the 3′-trailer and the mature tRNA sequences. The expected site for EndoV cleavage is also indicated (triangle) and the anticodon of the tRNA is underlined. (**B**) Chromatogram obtained after sequencing of the anticodon loop of *in vitro* transcribed pre-tRNA^Val^_(AAC)_ incubated ((+) hetADAT) or not ((−) hetADAT) with purified human hetADAT. (**C**) EndoV assay for detection of inosine on *in vitro* transcribed pre-tRNA^Val^_(AAC)_ that has been previously incubated ((+) hetADAT) or not ((−) hetADAT) with purified human hetADAT. Pre-tRNA^Val^ was ^32^P-radiolabelled after EndoV treatments allowing for detection of all the cleavage products. Expected tRNA lengths: 95 nt for full length pre-tRNA^Val^_(AAC)_; 54 nt for 3′-tRNA arm after EndoV cleavage if inosine is present at position 34 of the tRNA; 41 nt for 5′-tRNA arm after EndoV cleavage if inosine is present at position 34 of the tRNA. ^32^P-radiolabelled RNA decade marker was used to estimate the molecular sizes.

If I34 occurs early at the pre-tRNA level, it means that precursor tRNAs already have all the necessary features to be a substrate for hetADAT. We tested this hypothesis by *in vitro* transcribing a pre-tRNA^Val^_(AAC)_ and incubating it with purified human hetADAT. We designed the pre-tRNA^Val^_(AAC)_ sequence to mimic the sequences observed in the reads of our RNAseq experiments (Figure 2A). Human hetADAT was cloned from the human protein sequences annotated as ADAT2 and ADAT3 (see ‘Materials and Methods’ section). After incubation with hetADAT, we performed a reverse transcription PCR reaction of the pre-tRNA and sequenced the obtained product. As shown in Figure 2B, sequencing of the anticodon loop of untreated pre-tRNA^Val^_(AAC)_ resulted in the expected ‘A’ at position 34. On the contrary, in the case of hetADAT treated pre-tRNA^Val^_(AAC)_ a clear ‘G’, without any traces of ‘A’, was observed at this position, consistent with a full conversion of A-to-I upon hetADAT treatment.

It has recently been reported that EndoV, a DNA repair enzyme, is capable of specifically cleaving inosines on RNA (38,39); so we took advantage of this activity to further confirm the *in vitro* A-to-I conversion by hetADAT. Briefly, pre-tRNA^Val^_(AAC)_ incubated with hetADAT was incubated with EndoV. Following EndoV treatment, the obtained RNA products were dephosphorylated and 5′-radiolabelled with ^32^P. The radiolabelled RNA was loaded in a denaturing gel and the band patterns were examined. As shown in Figure 2C, a cleavage pattern was observed upon EndoV incubation only in the pre-tRNA previously treated with hetADAT. As expected for this tRNA, the full-length molecule was cleaved only at one position resulting into two bands. These two bands corresponded in size to the expected 5′ and 3′ tRNA halves that result from the cleavage by EndoV at an inosine at the first anticodon position (I34) of the tRNA (Figure 2).

We also tested the activity of hetADAT on a mature-like tRNA substrate. As shown in Supplementary Figure S6, human hetADAT could deaminate *in vitro* transcribed tRNA^Ala^_(AGC)_ specifically at position 34. This is in agreement with previous reports where other mature-like tRNA sequences were tested for A34 deamination (10–12,30). These results show that 5′-leader and 3′-trailer sequences are not an absolute requirement for hetADAT activity and further support the idea that pre-tRNAs are modified by hetADAT as they mature (Figure 1C and see above). Importantly, these results also show that, *in vitro*, hetADAT can deaminate A34 on tRNA^Ala^, but not A37 (which is believed to be modified to m^1^I37 by ADAT1 and Trm5) (35,36).

Altogether these results suggest that human precursor tRNAs can be substrates of hetADAT. They also suggest that no other post-transcriptional tRNA modifications are necessary for hetADAT activity, since we used for our assays an *in vitro* transcribed tRNA. These experiments also further validates that inosine is read as a ‘G’ when being sequenced and that the two genes annotated in NCBI as human ADAT2 and ADAT3 are in fact encoding for the subunits of human hetADAT.

### I34 modification is likely to occur in nucleus

**Figure 3.**
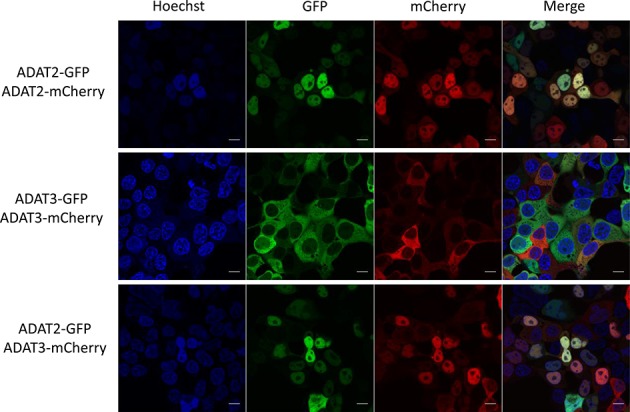
Live imaging confocal microscopy of HEK293T cells after co-expression of GFP- or mCherry-tagged HsADAT2 and HsADAT3 proteins. Scale bar corresponds to 10 μm. Shown are the results for C-term tagged ADAT proteins (see also Supplementary Figure S7).

Pre-tRNAs are believed to be processed mainly in nucleus (40). We therefore predicted that hetADAT should also be located in nucleus in order to modify pre-tRNAs. We decided to address this question by performing confocal microscopy. Unfortunately, we were unable to detect endogenous ADAT2 or ADAT3 by immunohistochemistry in human cell lines using commercially available antibodies so we chose to express fluorescently tagged proteins. To avoid misleading results, we cloned human ADAT2 and human ADAT3 as N-term or C-term fusion proteins with two different fluorophores: GFP and mCherry (eight constructs in total). By doing so, we could rule out potential localization artifacts due to the presence of a particular type of fluorophore or whether the fluorophore was fused to the N-term or the C-term of the protein. Additionally, expressing fluorescent proteins allowed us to perform live imaging, ruling out artifacts often observed by immunohistochemistry due to cell fixation protocols (41). As observed in Figure 3 (upper panel), GFP- or mCherry-tagged ADAT2 localized mainly to nucleus and partially to the cytoplasm. Surprisingly, GFP- or mCherry-tagged ADAT3 localized mainly to cytoplasm and only partially to nucleus (Figure 3 centre panel). Given that ADAT2 and ADAT3 are expected to work as a heterodimer, we thought that the localization might be dependent on the stoichiometry of the subunits. We therefore co-expressed GFP-tagged ADAT2 and mCherry-tagged ADAT3 and found both subunits co-localizing in nucleus (Figure 3 lower panel). Similar results were obtained with either combination of the eight different fluorescently-tagged proteins (Supplementary Figure S7). While it is nevertheless important to verify the subcellular localization of the endogenous ADAT2 and ADAT3; these results suggest that hetADAT acts mainly in nucleus and that ADAT3 translocates to nucleus in an ADAT2-dependent manner.

### Knockdown of ADAT2 reduces the levels of I34 in cells

HetADAT has never been studied in human cell lines. We therefore thought that a final validation of the results we have presented this far would be to deplete endogenous ADAT2 (the catalytic subunit of hetADAT) in cells and evaluate the levels of I34 modification by RNAseq. This would confirm that RNAseq is a suitable method to detect I34, as a lowering in I34 modification levels should be expected. Additionally, it will give further functional evidence that the human gene annotated as ADAT2 (NCBI, see ‘Materials and Methods’ section) is indeed the human homologue of the previously characterized yeast Tad2 (7,16), *T. brucei* ADAT2 (17–19) and *A. thaliana* ADAT2 (20).

**Figure 4.**
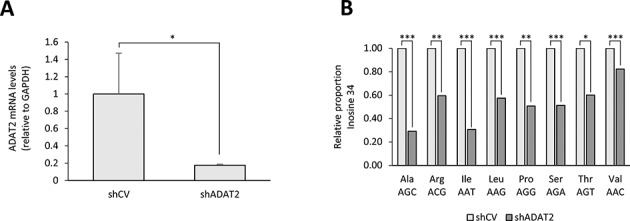
(**A**) RT-qPCR showing the levels of hADAT2 mRNA relative to the mRNA levels of GAPDH in HEK293T shCV cells and HEK293T shADAT2 cells. Shown is the average and standard deviation for three independent replicates. Statistical significance was obtained with a *t*-test (*: *P*-value ≤ 0.05). (**B**) Relative proportion of inosine found at position 34 in all human ANN tRNAs for HEK293T shCV cells and HEK293T shADAT2 cells. Statistical significance was obtained by Fisher Exact Test with Benjamini–Hochberg adjusted *P*-values (***: *P*-value ≤ 0.001; **: *P*-value ≤ 0.01; *: *P*-value ≤ 0.05).

We attempted to generate HsADAT2 KO cell lines using the CRISPR/Cas9 technology. Out of more than 100 HEK293T CRISPR/Cas9-treated single cells, only about 30% were viable and of those, no clone was a full ADAT2 KO (data not shown). These results suggest that in humans, just like for other eukaryotes (7,18,20), ADAT2 is probably an essential gene. We therefore generated HEK293T cells stably expressing a short-hairpin RNA (shRNA) against human ADAT2 (shADAT2) or expressing an shRNA with a non-target sequence (control vector: shCV). This strategy resulted in viable cells and, as shown in Figure 4A, ADAT2 mRNA levels were strongly downregulated in shADAT2 cells as compared with shCV cells. We next performed RNAseq experiments on both of these cell lines and found that for all eight possible tRNA substrates of hetADAT the levels of I34 modification were significantly reduced upon HsADAT2 knockdown (Figure 4B). We observed similar sequencing biases (mapping score, number, length and quality of the reads) towards different tRNA sequences in all our RNAseq experiments; which allow us to confidently compare the levels of A34 versus G34 (I34) for a given tRNA between shCV and shADAT2 cells (e.g. tRNA^Ile^ shCV versus tRNA^Ile^ shADAT2). However, given these sequencing biases, we believe it would be misleading to compare how the levels of I34 modification change among different types of tRNAs (e.g. the levels of I34 for tRNA^Ile^ seem far more reduced than the levels of I34 for tRNA^Val^; also see above). The non-normalized proportion values of I34 detected for each ANN tRNA can be seen in Supplementary Figure S8. Altogether, these results confirms that the HsADAT2 gene we have targeted is indeed encoding for a subunit of the human hetADAT and that RNAseq can be used to monitor the levels of a post-transcriptional tRNA modification occurring at the pre-tRNA level. This data also suggest that even though I34 is likely an essential tRNA modification in human cells, modulations in the levels of such modification can be tolerated in this species.

## DISCUSSION

RNAseq is a powerful tool to detect and quantify RNA species. However, the use of this technology to detect and quantify tRNAs has been hampered due to the difficulties in sequencing these type of RNAs (32–34). It has only been in the past few years that RNAseq protocols and bioinformatics analyses have been optimized for studying tRNAs and even so, until now, such strategies proved useful to answer only a particular set of biological questions. For example, by analysing the mapping of mismatches observed between sequencing reads and the genomic region where those reads mapped, known post-transcriptional base modifications on tRNAs and potentially novel modified tRNA residues could be detected on a wide range of species (31,33,42,43). Additionally, RNAseq proved useful to unravel the order in which precursor tRNAs are processed in the hyperthermophile *Methanopyrus kandleri* (44). More recently, a modification to the RNAseq protocol allowed for a strong quantitative analysis of tRNA levels and was used to assess the effect of different types of cellular stress on tRNA abundance in yeast (32).

By performing RNAseq experiments, we could detect with high confidence the presence of I34 (read as a G34) on all eight tRNA substrates of hetADAT in early precursors and to a higher extent, in precursors after 5′-leader and 3′-trailer removal (Figure 1). We further showed that *in vitro*, a pre-tRNA can be a substrate of hetADAT using two different techniques: sequencing of pre-tRNA^Val^_(AAC)_ after incubation with hetADAT; and a novel EndoV-based assay to confirm that the modification observed by sequencing as a G34 is in fact an I34 (Figure 2). Moreover, given that pre-tRNAs are likely to be in nucleus, we expected hetADAT also to localize to nucleus and indeed we demonstrated that fluorescently-tagged ADAT2 and ADAT3 localized in this subcellular compartment (Figure 3). Finally, we have for the first time knocked down human ADAT2 and showed that the levels of I34 modification were reduced in all eight possible substrates of hetADAT as observed by RNAseq (Figure 4), validating this technique to study A-to-I editing at the pre-tRNA level.

While in the past the A34-to-I34 modification has been studied at the mature tRNA level, we found some evidence in the literature for this modification occurring at the pre-tRNA level. Achsel and Gross *in vitro* transcribed pre-tRNA^Val^_(AAC)_ chimeras containing the long variable arm of tRNA^Ser^, which were efficiently processed to mature tRNAs and deaminated to I34 upon HeLa nuclear extract incubation. However, their results did not rule out that it was the pre-tRNA^Val^ chimeras in fact the ones that were being deaminated by hetADAT and not the mature tRNA^Val^ (14). In light of our results, it seems more likely that indeed the pre-tRNA was the one being deaminated (Figure 2). More direct evidence for I34 on pre-tRNAs was reported by French and Trewyn. The authors generated a 195-nt long pre-tRNA-like transcript corresponding to *Bombix mori* tRNA^Ala^. They observed A34-to-I34 editing upon incubation of the pre-tRNA with whole cell extracts from human leukaemia cells without observing processing of the pre-tRNA (13). These two reports are in agreement with our findings and here we are further showing that not only synthetic pre-tRNAs can be modified by hetADAT *in vitro* but also natural ANN pre-tRNAs are modified into INN pre-tRNAs in living cells (Figures 1B and 2).

Many tRNA modifications have been reported to act at the pre-tRNA level and sometimes in a carefully ordered manner (40). For example, 5-methylcytosine at position 40 of yeast tRNA^Phe^_(GAA)_ can only occur before the splicing of the tRNA intron; while other modifications such as 2’-O-methylcytidine at position 32, 2’-O-methylguanosine at position 34 and 1-methylguanosine at position 37 on the same tRNA require a spliced tRNA (45). More recently, *Gaston et al*. showed that C-to-U editing at position 32 of *T. brucei* tRNA^Thr^ occurs prior to 5′-leader maturation and likely following 3′-trailer removal. Further, they showed that in this particular organism, C-to-U editing at position 32 stimulates but is not required for A-to-I editing at position 34 (19). We found indications of a stepwise process for the A37 modification on tRNA^Ala^_(AGC)_; where A37 is first deaminated to I37 followed by a further methylation to m^1^I37 (Supplementary Figure S5). This is consistent with published biochemical data (35,36). Additionally, in *A. thaliana*, ADAT1 was found localizing to nucleus, consistent with a role in A37-to-I37 modification at the pre-tRNA level (6). The subcellular localization of Trm5 has been addressed in *T. brucei*, where it was found to localize in nucleus and cytoplasm (46). Our data suggests that Trm5 would also be acting at the pre-tRNA level although preferably after 5′-leader and 3′-trailer removal (Supplementary Figure S5). Regarding I34 formation, our data shows that this modification does not require 5′-leader or 3′-trailer removal (Figure 1B ‘precursor’ group and Supplementary Figure S2). This was also reported by French and Trewyn *in vitro* (13). Additionally, in agreement with a previous report (11), we showed *in vitro* that no other tRNA post-transcriptional modifications are required for hetADAT activity (Figure 3); consistent with hetADAT recognizing its substrates early in the biogenesis of tRNAs. The fact that A34-to-I34 editing starts to occur before 5′-leader and 3′-trailer removal and that a significant increase on I34 modification can be observed in reads that are lacking the leader and trailer sequences (Figure 1B) might suggest that the modification only occurs before precursor-end trimming. However, we find this unlikely given that some reads lacking leader and trailer sequences were found not modified and it is believed that when ANN tRNAs are fully matured, they are found nearly fully modified to I34 (29). Additionally, we showed that hetADAT can modify a mature-like tRNA substrate *in vitro*, suggesting that 5′-leader and 3′-trailer sequences are not required for hetADAT activity (Supplementary Figure S6). We therefore favour the hypothesis that hetADAT acts on pre-tRNAs as they maturate. As a result, the further downstream on the tRNA biogenesis process the more events of A34-to-I34 editing can be observed. Importantly, we cannot rule out the possibility that a proportion of A34 deamination can also still occur at the mature tRNA level (see also below).

It has been thoroughly demonstrated that hetADAT requires a specific tRNA structure in order to recognize and modify its substrates (10–12,14,30). While hetADAT was capable of modifying full length *E. coli* tRNA^Arg^, it was unable to modify an *E. coli* tRNA^Arg^ mini-substrate composed of only the tRNA^Arg^ anticodon stem loop (10,30). Disruption of typical 3D base-pairs (i.e. residues G19:C56, A15:U48, A14:U8) as well as extending the anticodon loop of a tRNA^Asp^_(AGC)_ mutant completely abolished A34-to-I34 editing (11). Likewise, I34 formation was also eliminated upon a single nucleotide deletion that severely alters the orientation of the variable arm on a synthetic pre-tRNA^Val^ (13). Given that we found natural pre-tRNAs modified with I34, the data suggests that pre-tRNAs already have the necessary tRNA structure for hetADAT substrate recognition. Additionally, the requirement for proper tRNA folding suggests that the tRNA fragments we detected containing I34 are not direct substrates of hetADAT, but rather the result of the processing of previously modified tRNA species (Figure 1).

The subcellular localization of hetADAT has been controversial. Grosjean *et al*. have used in the past an *in vitro* system to detect A34-to-I34 modification by injecting synthetic ANN tRNA chimeras into *Xenopus laevis* oocytes (12). They could observe efficient A34-to-I34 conversion and suggested that the modification occurred in the cytosol because it was unlikely that there was transfer of the chimeric tRNA from the cytoplasm to the nucleus (12). The same group also showed A34-to-I34 editing of a chimeric tRNA using cell extracts from anucleated rabbit reticulocytes (11). These two reports pointed towards the I34 modification occurring in cytoplasm in two highly specialized cellular types: oocytes and reticulocytes. The same seemed to be true in the lower eukaryote *T. brucei*, as two tRNA substrates of hetADAT (tRNA^Thr^_(AGU)_ and tRNA^Val^_(AAC)_) were not deaminated when incubated with nuclear extracts of *T. brucei* (19). However, Achsel and Gross showed that tRNA^Val^_(AAC)_ chimeras were capable of being deaminated to I34 when incubated with HeLa nuclear extracts (14). Moreover, in yeast, Tad2 and Tad3 were found to localize both in nucleus and cytoplasm as annotated in the Yeast GFP Fusion Localization Database (http://yeastgfp.yeastgenome.org/).

Our results suggest that hetADAT is acting in nucleus in HEK293T cells (Figure 3 bottom panel). In this regard, we have ruled out potential localization artifacts related to the chemistry of the fluorophore or its position in ADAT2 or ADAT3 (Supplementary Figure S7). Similarly, sample preparation artifacts were avoided with live cell imaging. However, our results have the caveat that in order to detect human ADAT2 and ADAT3 we had to overexpress the proteins. Attempts to detect endogenous ADAT2 or ADAT3 in HeLa cells by immunohistochemistry using commercially available antibodies were also unsuccessful (data not shown). Therefore, we cannot rule out the possibility that some A34-to-I34 modification might be occurring in the cytosol.

Our experiments with fluorescent ADAT2 and ADAT3 indicate that the nuclear localization of ADAT3 is dependent on ADAT2. Indeed, we found that ADAT3 was mainly localized in the cytoplasm, but translocated to nucleus in an ADAT2-dependent manner (Figure 3). This difference in individual localization of the subunits might indicate independent functional roles of ADAT2 and/or ADAT3, or might serve as a mechanism for hetADAT regulation in cells. We are currently investigating different biological scenarios where the interaction between the hetADAT subunits might be modulated and which could be the consequences of such regulation over protein translation.

In this work we also validated two genes annotated in NCBI as HsADAT2 and HsADAT3 as the ones encoding for the two subunits of the human hetADAT. First, we showed that the purified ADAT2 and ADAT3 proteins, together, are capable of specifically deaminating A34 to I34 on a tRNA *in vitro* (Figure 2 and Supplementary Figure S6). Secondly, we knocked down human ADAT2 and showed, for the first time, that the levels of I34 can be modulated on all eight human tRNA substrates of hetADAT (Figure 4). This result also allowed us to further validate our RNAseq experiments as an efficient method to study tRNA modifications at the pre-tRNA level. Since we were unable to obtain full ADAT2 KO cells, we believe that this gene is essential in this species, just as previously reported for yeast (7), *T. brucei* (18) and *A. thaliana* (20). However, our shADAT2 cell lines were viable suggesting that human cells can tolerate some degree of modulation of the I34 modification; as previously reported for yeast (16), *T. brucei* (18) and *A. thaliana* (20).

Given the importance of inosine in codon:anticodon interactions, the tolerance to inosine variations may reflect a role for this modification in the control of gene translation. We have previously shown that highly expressed genes are enriched in codons recognized by ADAT-modified tRNAs (ADAT-preferred codons) (9). We also found that mRNAs enriched in ADAT-preferred codons, but not those depleted of ADAT-preferred codons, are more prone to be associated to specific biological functions as the organism complexity increases (3). It is possible that human cells could tolerate some variations in the levels of I34 modification because only a subset of transcripts (i.e. those highly expressed and enriched in ADAT-preferred codons), and only a subset of biological functions (i.e. those involving key proteins encoded by mRNAs with a high ADAT-preferred codon enrichment), may be affected by this process. This possibility is in agreement with recent analyses of ribosome profiling data in yeast, which revealed that the correlation between codon bias and translation efficiency is the result of the selection of codons to exploit efficiently the components of the translation machinery in highly translated genes (47). In this scenario, a controlled modulation of I34 levels could limit or promote the availability of optimal INN tRNAs and could serve as an additional layer of regulation for specific genetic programs in the cell. This does not exclude that inosine content in tRNA anticodons may be essential for the synthesis of proteins with an extremely biased composition toward amino acids that can use ADAT-preferred codons.

**Figure 5.**
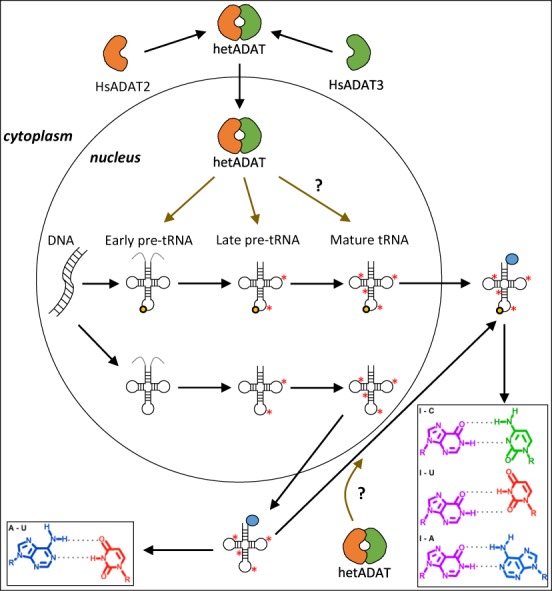
A proposed model for the incorporation of I34 into human tRNAs. Leader and trailer sequences on pre-tRNAs are shown in light grey colour. I34 modification is depicted as a light orange circle at position 34 of the tRNA; while other tRNA modifications are shown as red asterisks. Amino acids on mature tRNAs are illustrated as a blue circle at the 3′-end of the tRNA molecule. Question marks indicate speculative processes. Brown arrows indicate hetADAT activity on different tRNA substrates. Nucleobase pairing for I34 (purple chemical structure) and A34 (blue chemical structure) are shown. See main text for full details.

We can now propose a model for the biogenesis of I34 in human tRNAs (Figure 5). Since HsADAT3 translocates from the cytoplasm to the nucleus in a HsADAT2-dependent manner, it is possible that the interaction between these subunits occurs in cytoplasm and is followed by its translocation to the nucleus. Once in the nucleus, hetADAT targets ANN pre-tRNAs as they mature. It is also possible that a proportion of I34 modification also occurs in cytoplasm. The resulting I34-containing tRNAs would now have an expanded codon decoding capacity (as compared to unmodified A34 tRNAs) due to inosine ‘wobble’ pairing, that may affect the translation of transcripts involved in particular genetic programs (3,48).

## SUPPLEMENTARY DATA

Supplementary Data are available at NAR Online.

SUPPLEMENTARY DATA

## ACCESSION NUMBERS

EBI European Nucleotide Archive, Accession number PRJEB8019.
