# Supplementary material for: Inosine modifications in human tRNAs are incorporated at the precursor tRNA level
Source: Nucleic Acids Res. 2015 Apr 27;43(10):5145–57. doi: 10.1093/nar/gkv277 (PMC4446420; doi:10.1093/nar/gkv277)
Supplement: SUPPLEMENTARY DATA [file supp_43_10_5145__index.html]

Inosine modifications in human tRNAs are incorporated at the precursor tRNA level — SUPPLEMENTARY DATA 

# Inosine modifications in human tRNAs are incorporated at the precursor tRNA level

## SUPPLEMENTARY DATA

- SUPPLEMENTARY DATA
