# Supplementary material for: Inosine modifications in human tRNAs are incorporated at the precursor tRNA level
Source: Nucleic Acids Res. 2015 Apr 27;43(10):5145–57. doi: 10.1093/nar/gkv277 (PMC4446420; doi:10.1093/nar/gkv277)

***Torres et al.* Inosine modifications in human tRNAs are incorporated at the precursor-tRNA level**

Supplementary Figures and Table.

Supplementary Table 1. Oligonucleotides (DNA oligos) used in this study.

Oligo	Sequence (5'-3')
pre-tRNA Val AAC oligo 1	AGCTTAATACGACTCACTATAGGGTTGTTTCCGTAGTGT
pre-tRNA Val AAC oligo 2	AGTGGTTATCACGTTCGCCTAACACGCGAAAGGTCCCTG
pre-tRNA Val AAC oligo 3	GATCAAAACCAGGCGGAAACAAGTGGTTACCCTTCTTACATGTG
pre-tRNA Val AAC oligo 4	ATAACCACTACACTACGGAAACAACCCTATAGTGAGTCGTATTA
pre-tRNA Val AAC oligo 5	TTTTGATCCAGGGACCTTTCGCGTGTTAGGCGAACGTG
pre-tRNA Val AAC oligo 6	GATCCACATGTAAGAAGGGTAACCACTTGTTTCCGCCTGG
Forward primer HsADAT2 In-Fusion pPEU17 (ADAT2-NtermCherry)	AAGTTCTGTTTCAGGGCCCGGAGGCGAAGGCGGCACCCA
Reverse primer HsADAT2 In-FusionpPEU17 (ADAT2-NtermCherry)	ATGGTCTAGAAAGCTTTAAGATTTCTGACATTCCTTTTTCCGAAC
Forward primer HsADAT2 In-Fusion pPEU19 (ADAT2-CtermCherry)	AGGAGATATACCATGGAGGCGAAGGCGGCACCCAA
Reverse primer HsADAT2 In-Fusion pPEU19 (ADAT2-CtermCherry)	CTTCCAGACCGCTTGAAGATTTCTGACATTCCTTTTTCCGAAC
Forward primer HsADAT3 In-Fusion pPEU17 (ADAT3-NtermCherry)	AAGTTCTGTTTCAGGGCCCGATCCTCTGCTCCCGTCTCTGTCT
Reverse primer HsADAT3 In-Fusion pPEU17 (ADAT3-NtermCherry)	ATGGTCTAGAAAGCTTTACGTGTCGGGGTCCAGCCAGCG
Forward primer HsADAT3 In-Fusion pPEU19 (ADAT3-CtermCherry)	AGGAGATATACCATGATCCTCTGCTCCCGTCTCTGTCT
Reverse primer HsADAT3 In-Fusion pPEU19 (ADAT3-CtermCherry)	CTTCCAGACCGCTTGACGTGTCGGGGTCCAGCCAGCG
Forward primer HsADAT2 pEGFP-N1 (ADAT2-CtermGFP)	TTATAAGCTTATGGAGGCGAAGGCGGCA
Reverse primer HsADAT2 pEGFP-N1 (ADAT2-CtermGFP)	AACTGGTACCCAAGATTTCTGACATTCCTTTTTCCG
Forward primer HsADAT2 pEGFP-C1 (ADAT2-NtermGFP)	TTATAAGCTTTGGAGGCGAAGGCGGCAC
Reverse primer HsADAT2 pEGFP-C1 (ADAT2-NtermGFP)	TATTGGTACCTCAAGATTTCTGACATTCCTTTTTCC
Forward primer HsADAT3 pEGFP-N1 (ADAT3-CtermGFP)	ATATAAGCTTATGATCCTCTGCTCCCGTCT
Reverse primer HsADAT3 pEGFP-N1 (ADAT3-CtermGFP)	TATAGGTACCGTGTCGGGGTCCAGCC
Forward primer HsADAT3 pEGFP-C1 (ADAT3-NtermGFP)	ATATAAGCTTTGATCCTCTGCTCCCGTCTC
Reverse primer HsADAT3 pEGFP-C1 (ADAT3-NtermGFP)	TATAGGTACCTACGTGTCGGGGTCCAG
Forward primer RT-qPCR HsADAT2	AAGGCTGAGATTGGAAGCTG
Reverse primer RT-qPCR HsADAT2	AGCAATTCACCATGAGCTGT
tRNA Ala AGC oligo 1	AGCTTAATACGACTCACTATAGGGGGTGTGGCT
tRNA Ala AGC oligo 2	CAGTGGTAGAGCGCGTGCTTAGCATGCACGAG
tRNA Ala AGC oligo 3	GCCCCGGGTTCAATCCCCGGCACCTCCACCAGGG
tRNA Ala AGC oligo 4	CGCTCTACCACTGAGCCACACCCCCTATAGTGAGTCGTATTA
tRNA Ala AGC oligo 5	TTGAACCCGGGGCCTCGTGCATGCTAAGCACG
tRNA Ala AGC oligo 6	GATCCCCTGGTGGAGGTGCCGGGGA

Supplementary Figure 1. Proportion (%) of tRNA reads corresponding to the Precursor tRNA group (left panel) or the Processed tRNA group (right panel) for every tRNA type detected by RNAseq in our study. Replicates correspond to 4 independent RNAseq experiments.

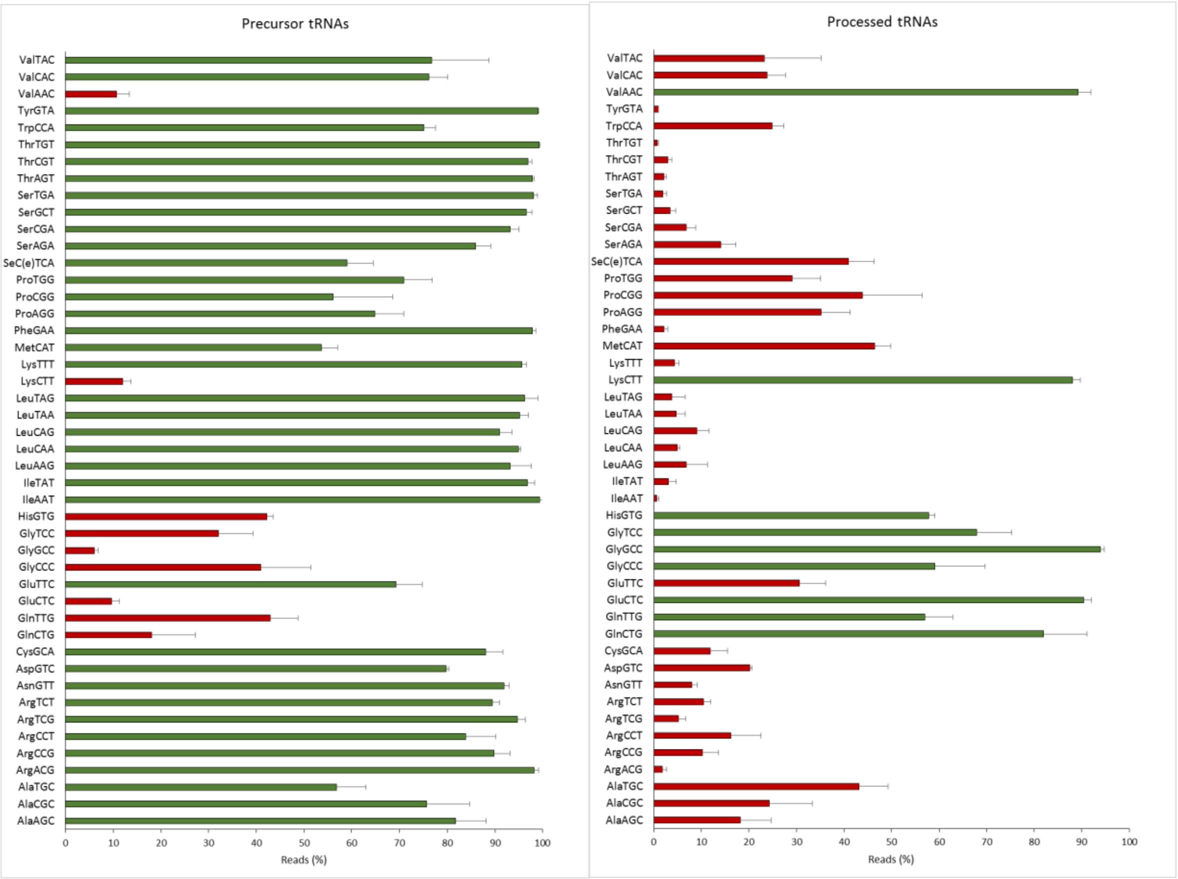

Supplementary Figure 2. Proportion (%) of bases found at position 34 for tRNA Ile AAT and tRNA Thr AGT within the “Precursor” tRNA group. In both cases inosine 34 (read as a G34) could be detected in the “Precursor” tRNA group. The “Processed” tRNA group was not analyzed for these tRNAs because of low read counts. We obtained less than 30 reads for “Processed” tRNA Ile AGT and less than 10 reads for “Processed” tRNA Thr AGT.

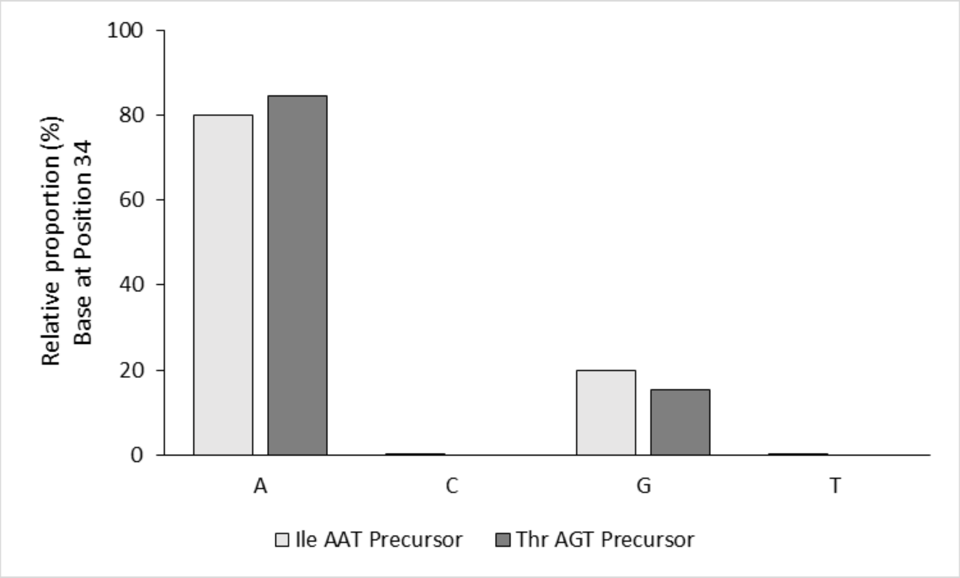

Supplementary Figure 3. The SeqLogo package version 1.24.0 was used to generate sequence logos showing nucleotide proportions around anticodon+1 position for tRNAs Ile(AAT), Leu(AAG) and Val(AAC). Shown are the results for base calls considering reads from the "Precursor tRNA" and "Processed precursor tRNA" groups together, for each tRNA type. While A-to-I deamination is observed at position A34 (detected as G34), no deamination is detected at adenosines in position 35 (A35) in these tRNAs.

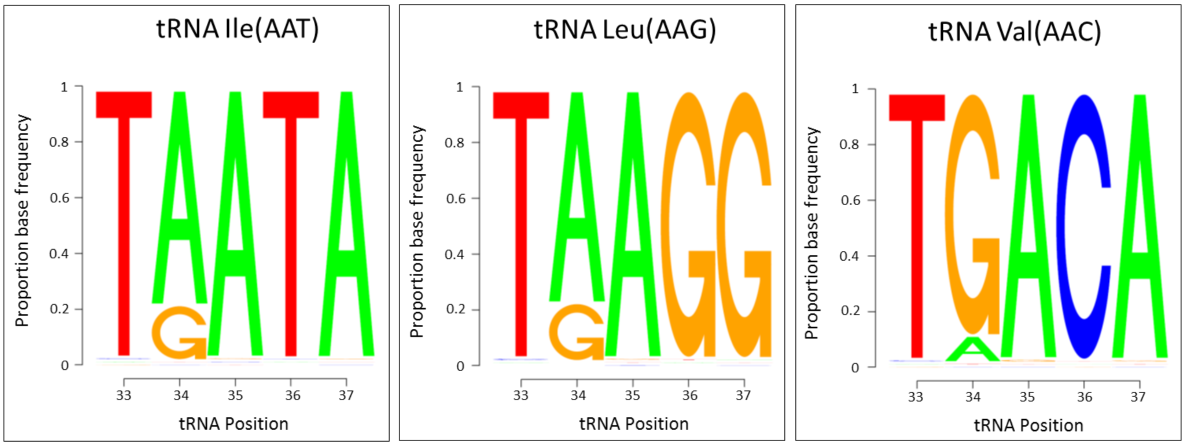

Supplementary Figure 4 (see next page). A-to-G mismatch proportion observed by RNAseq at residues throughout the whole sequence of the tRNA for all human ANN tRNAs. Background/noise levels were defined as the signal below 1% of the A-to-G mismatch error rate and is represented in the graphs as a grey dashed line. Residues showing an A-to-G mismatch proportion higher than 1% are indicated. Pos34: I34; Pos37: I37 (will be further modified to m^1I37); Pos58: likely corresponds to m^1A (m^1A detected as a "G" when sequenced has been reported previously (30, 41, 42)). Note that each graph is depicted using different scales at the y-axes showing the strong sequencing biases towards detection of the A-to-G mismatch proportion in different tRNA types.

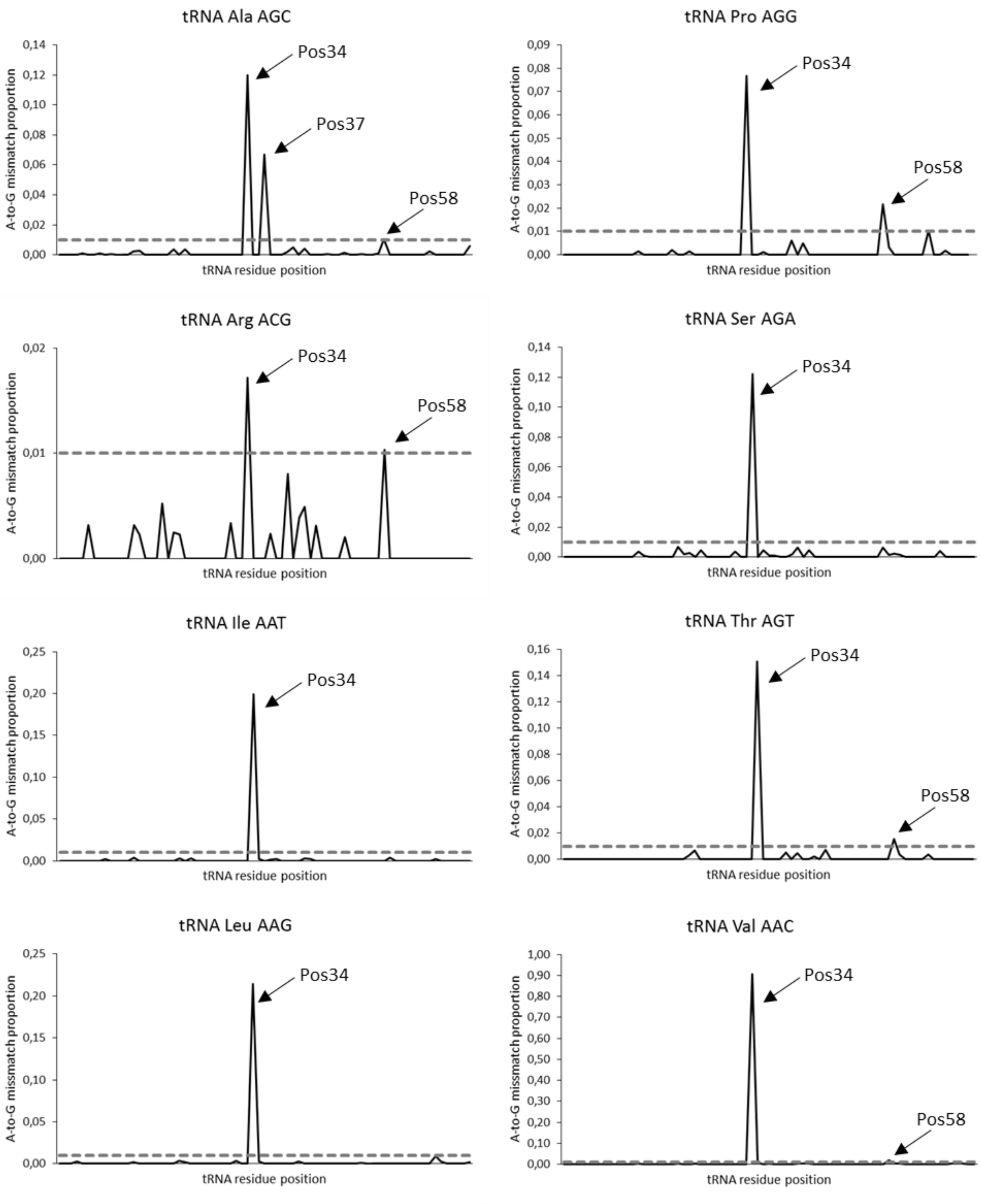
tRNA Ala AGC
0,14
0,12
0,10
0,08
0,06
0,04
0,02
0,00
Pos34
Pos37
Pos58
A-to-G mismatch proportion
tRNA residue position
tRNA Pro AGG
0,09
0,08
0,07
0,06
0,05
0,04
0,03
0,02
0,01
0,00
Pos34
Pos58
A-to-G mismatch proportion
tRNA residue position
tRNA Arg ACG
0,02
0,01
0,00
Pos34
Pos58
A-to-G mismatch proportion
tRNA residue position
tRNA Ser AGA
0,14
0,12
0,10
0,08
0,06
0,04
0,02
0,00
Pos34
A-to-G mismatch proportion
tRNA residue position
tRNA Ile AAT
0,25
0,20
0,15
0,10
0,05
0,00
Pos34
A-to-G mismatch proportion
tRNA residue position
tRNA Thr AGT
0,16
0,14
0,12
0,10
0,08
0,06
0,04
0,02
0,00
Pos34
Pos58
A-to-G mismatch proportion
tRNA residue position
tRNA Leu AAG
0,25
0,20
0,15
0,10
0,05
0,00
Pos34
A-to-G mismatch proportion
tRNA residue position
tRNA Val AAC
1,00
0,90
0,80
0,70
0,60
0,50
0,40
0,30
0,20
0,10
0,00
Pos34
Pos58
A-to-G mismatch proportion
tRNA residue position

Supplementary Figure 5. Proportion (%) of bases found at position 37 for tRNA Ala AGC. Only A37 (unmodified A37) and G37 (I37) could be detected in the Precursor tRNA group. In the Processed tRNA group higher levels of G37 (I37) and T37 (m^1I37) could be detected.

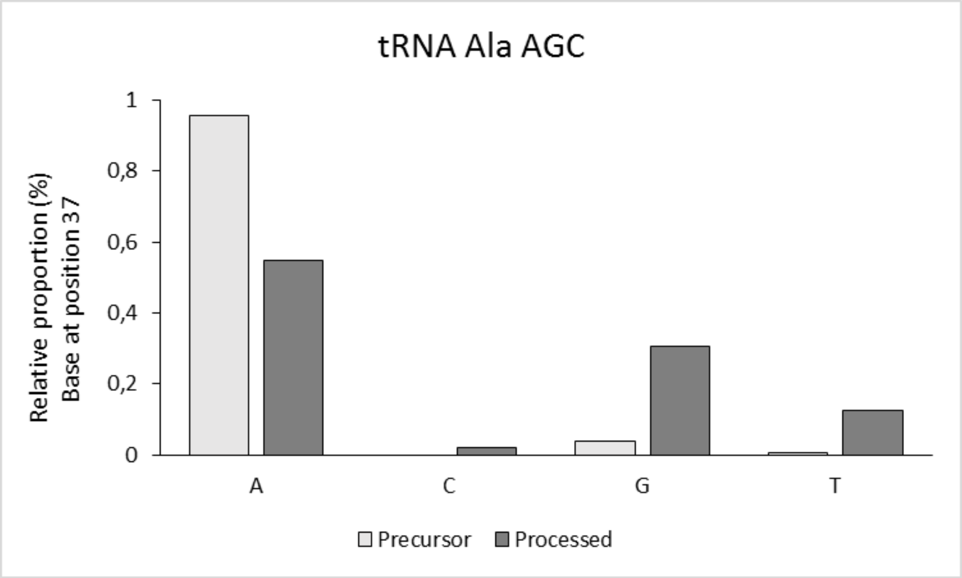

Supplementary Figure 6. Chromatogram obtained after sequencing of the anticodon loop of *in vitro* transcribed $tRNA^{Ala}{}_{(AGC)}$ incubated ((+) hetADAT) or not ((-) hetADAT) with purified human hetADAT. The experiment was carried out exactly as described for $pre\text{-}tRNA^{Val}{}_{(AAC)}$ in **Figure 2**. As expected, A37 was not deaminated by hetADAT.

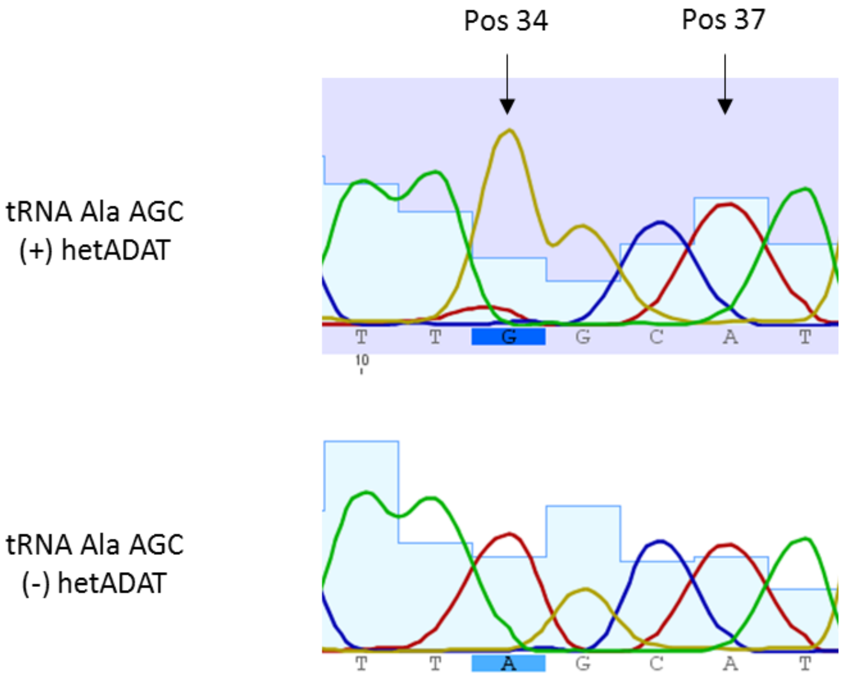

Supplementary Figure 7. Live imaging confocal microscopy of HEK293T cells following co-expression of GFP- or mCherry-tagged HsADAT2 and HsADAT3 proteins. Scale bar corresponds to 5 µm. Shown are the results for all the combinations of C-term tagged or N-term tagged ADAT proteins not shown in **Figure 3**.

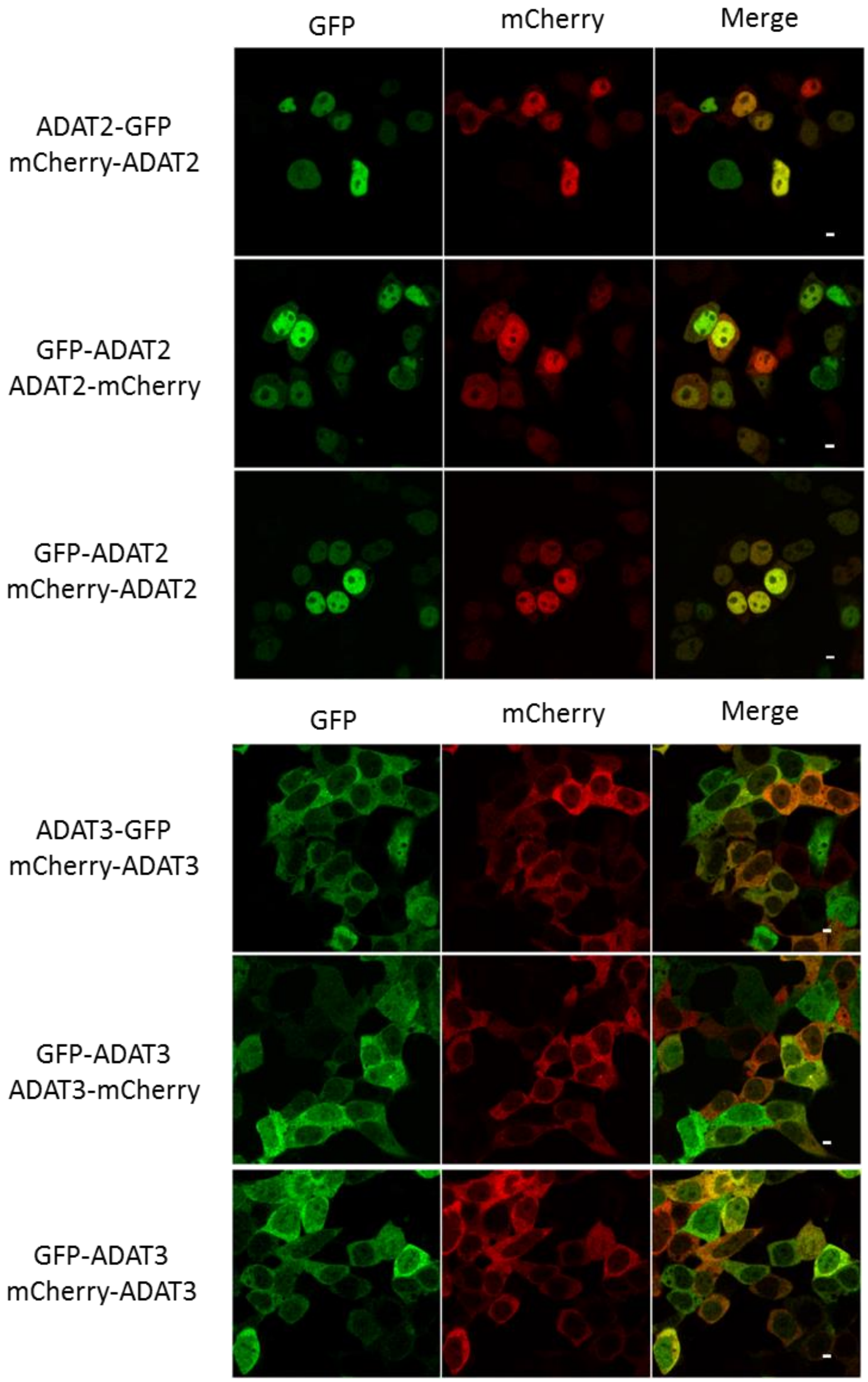

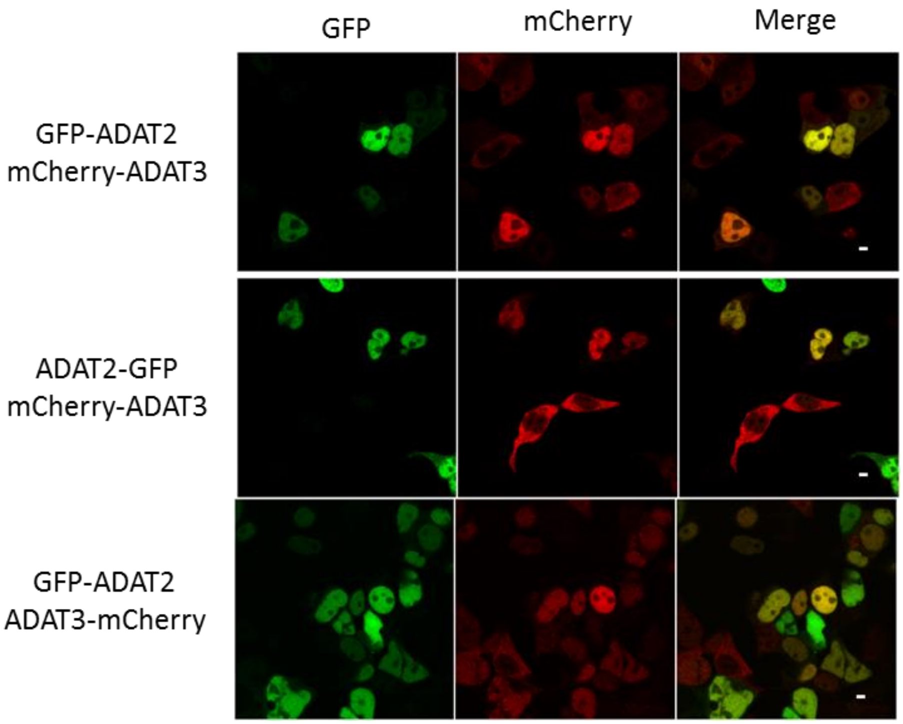
GFP
mCherry
Merge
GFP-ADAT2
mCherry-ADAT3
ADAT2-GFP
mCherry-ADAT3
GFP-ADAT2
ADAT3-mCherry

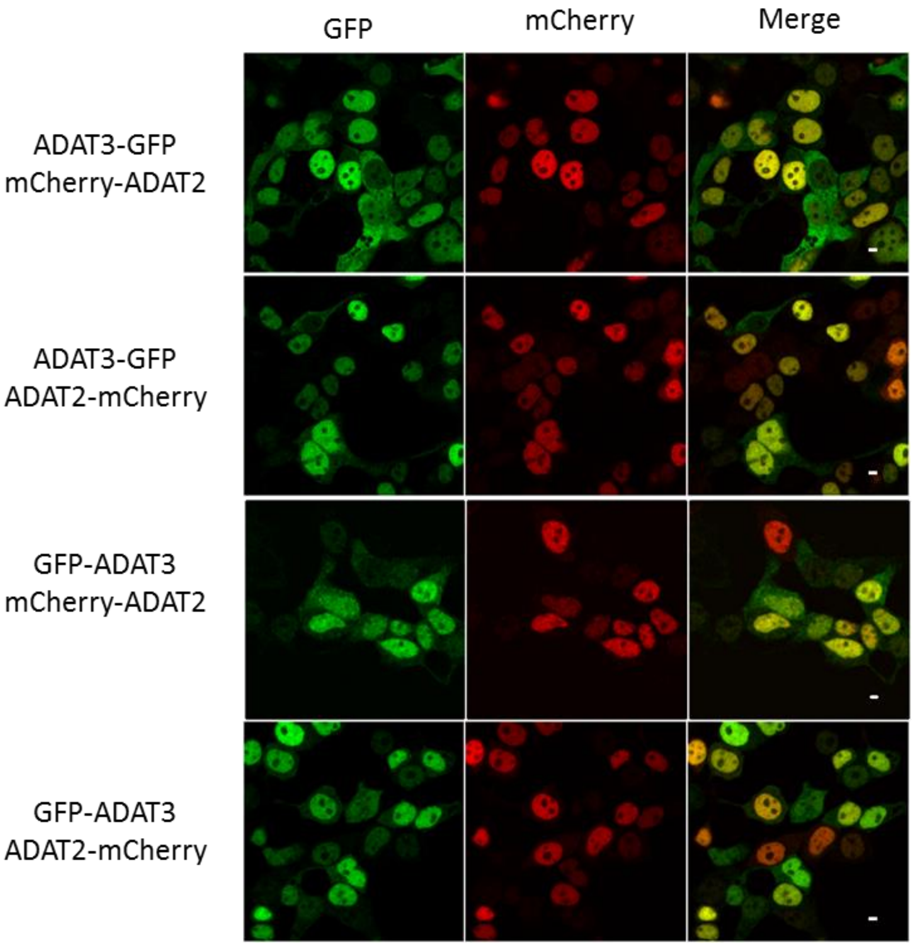
GFP
mCherry
Merge
ADAT3-GFP
mCherry-ADAT2
ADAT3-GFP
ADAT2-mCherry
GFP-ADAT3
mCherry-ADAT2
GFP-ADAT3
ADAT2-mCherry

Supplementary Figure 8. Non-normalized proportion (%) of I34 observed in HEK293T shCV cells and HEK293T shADAT2 cells for all human ANN tRNAs. Figure corresponds to the non-normalized values of **Figure 4B**.

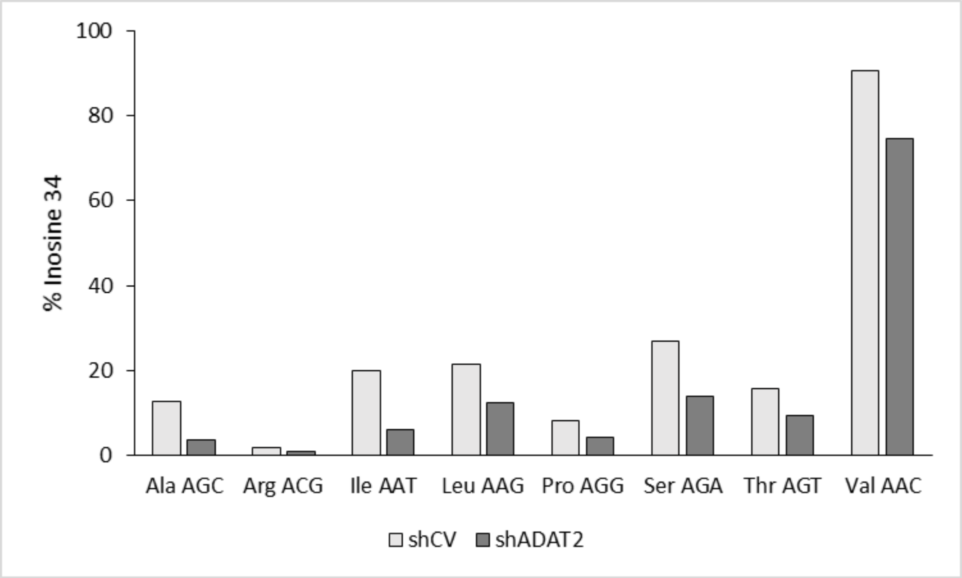

Supplement: SUPPLEMENTARY DATA [file supp_gkv277_nar-00204-r-2015-File008.pdf]
